# Oregano Feed Supplementation Affects Glycoconjugates Production in Swine Gut

**DOI:** 10.3390/ani10010149

**Published:** 2020-01-16

**Authors:** Francesca Mercati, Cecilia Dall’Aglio, Gabriele Acuti, Valerio Faeti, Federico Maria Tardella, Carolina Pirino, Elena De Felice, Paola Scocco

**Affiliations:** 1 Department of Veterinary Medicine, University of Perugia, Via San Costanzo 4, 06126 Perugia, Italy; francesca.mercati@unipg.it (F.M.); gabriele.acuti@unipg.it (G.A.); carolpirino@alice.it (C.P.); 2 Council for Agricultural Research and Economics, Research Centre for Animal Production and Aquaculture, Modena Unit, Via Beccastecca, 345, 41018 San Cesario sul Panaro, Italy; valerio.faeti@crea.gov.it; 3 School of Biosciences and Veterinary Medicine, University of Camerino, Via Pontoni 5, 62032 Camerino, Italy; dtfederico.tardella@unicam.it (F.M.T.); paola.scocco@unicam.it (P.S.)

**Keywords:** swine, gut glycoconjugates, *Origanum vulgare* L., BAX, oxidative stress

## Abstract

**Simple Summary:**

The European ban towards antibiotics is increasing the number of studies on the effects of feed additives, such as plant extracts, in order to enhance the health and welfare status of domestic animals intended for human consumption. *Origanum vulgare* possesses multiple pharmacological characteristics and its antioxidant and antibacterial properties are particularly interesting. Besides, a recent study aimed at evaluating the effects of oregano aqueous extract supplementation in poultry nutrition gave encouraging results regarding the secretion of glycoconjugates in the gut which increases tissue hydration and protects the intestinal mucosa from pathogenic bacteria, viruses, and parasites. Therefore, we investigated the effects of oregano feed supplementation on antioxidant and defense ability of pig gut. Our results showed that there was improved production of glycoconjugates in the duodenum and colon of pigs fed with supplementation of oregano aqueous extract, enhancing protection of the mucosa of these sections of the intestine. Also, we observed an enhanced antioxidant action in the two examined gut tract samples of the group supplemented with oregano. Findings can be used in further research to identify ways to improve endogenous defense ability with to reduce antibiotic use and prevent antimicrobial resistance.

**Abstract:**

This study evaluated the effects of adding oregano aqueous extract (OAE) to the diet of pig slaughtered at finisher stage. Study was performed to identify glycoconjugates and evaluate the oxidative stress levels in the duodenum and colon intestinal tracts. Glycohistochemistry was performed by staining with Periodic acid–Schiff (PAS), Alcian blue (AB) pH 2.5, AB-PAS, AB pH 1, AB pH 0.5, low iron diamine, and high iron diamine. Serial sections were pre-treated with sialidase V before staining with AB pH 2.5 (Sial-AB) preceded or not by saponification. To study oxidative stress, an immunohistochemical analysis was applied to investigate the presence of the oxidative stress target molecule Bcl-2 Associate X protein (BAX). Findings show that oregano aqueous extract supplementation improves the production of the secretion glycoconjugates involved in direct and indirect defense, thus enhancing the protection of the pig intestinal mucosa. Moreover, the reduced BAX protein immunostaining observed in both duodenum and colon of swine of the oregano-supplemented group respect to that observed in the control group suggests an enhanced antioxidant action by oregano adding. Findings could be useful for other studies aiming to reduce antibiotic use and prevent antimicrobial resistance.

## 1. Introduction

The European ban towards antibiotics has increased the number of studies on the effects of feed additives—such as plant extracts, trace minerals, organic acids, probiotics, and polyunsaturated fatty acids [1,2,3,4,5,6]—in order to enhance the health and welfare status of domestic animals intended for human consumption. Moreover, a significant correlation between oxidative status, productive performance, and gastrointestinal mucosal disease was observed in intensive rearing systems, such as poultry and pig farms [7,8]. Oxidative stress is a pathological condition caused by alterations in the balance between the production and the elimination of reactive oxygen species by the organism [9]. The accumulation of reactive oxygen species can damage biomolecules (such as lipids, proteins, and DNA) and alter their normal function leading to programmed cell death. Besides causing cellular damage, oxidative stress predisposes the individual to several pathological conditions and chronic disorders [10]. Endogenous antioxidant systems include both enzymatic and non-enzymatic antioxidants and are usually effective in counterbalancing the effects of oxidants and preventing their harmful effects. In addition, the agro-food and feed manufacturing industry has recently shown an increasing interest in plant extracts (i.e., thyme, rosemary, oregano, and other spices) as they are profitable sources of bioactive compounds [11]. *Origanum vulgare*, native to Europe and Western Asia, possesses multiple pharmacological characteristics such as antioxidant, antibacterial, antimycotic, and anti-inflammatory properties [12,13]. The antioxidant effect of this herb is attributed to the presence of two of its essential components: carvacrol and rosmarinic acid [12] and its vitamin C content [13]. Rosmarinic is the most represented polyphenol in *Origanum vulgare* [14], but there are counteracting data about its antioxidant activity [15]. Even if the Ferulic and *p*-Coumaric acids characterize the polyphenolic profile of oregano [14], its high caffeic acid content is responsible for its antioxidant activity; indeed, a correlation between total phenolic compounds and 1,10-diphenyl-2-picrylhydrazyl radical scavenging activity with the presence in the aqueous extract of *Origanum vulgare* of caffeic acid, eriodictyol, and apigenin was demonstrated [16]. The antimicrobial activity of oregano can be attributed to the presence of phenols, terpenes, aldehydes, and ketones that principally performed against the cell cytoplasmic membrane of microorganism. Different studies showed that oregano creates permeability in the cell membrane of microorganism: the increase in permeabilization provoked a decrease of the ATP concentration in the cells, a decrease of intracellular pH, and a release of the cell constituents, with the final death of cell [17,18].

A recent study aimed at evaluating the effects of oregano aqueous extract (OAE) supplementation in poultry nutrition found encouraging results regarding the secretion of glycoconjugates in the gut which increases tissue hydration and protects the intestinal mucosa from pathogenic bacteria, viruses, and parasites [19]. In fact, glycosaminoglycan-like materials form a mucous layer negatively charged on the free epithelium surface that preserve the hydration degree of mucous membrane and, in the first intestinal tract, protect it from the acidity of gastric material [20,21,22]. In addition, direct and indirect protective actions on mucous membranes are performed by a great variety of glycoconjugates secreted by different anatomical structures in many animal species [23,24,25,26,27] by means of mucopolysaccharides and/or sialoderivatives able to resist to the action of specific bacterial enzymes as hyaluronidase or neuraminidase [25,27,28,29,30,31]. Therefore, it is very interesting to investigate the modification in their secretion, as induced by bioactive compounds. This study aims to identify the glycoconjugates by means of glycohistochemical techniques able to characterize complex carbohydrates, and to evaluate the oxidative stress levels by immunohistochemical detection of a target molecule Bcl-2 associate X protein (BAX) in the duodenum and colon of pigs fed a diet supplemented with OAE, in order to validate the antioxidant action of oregano and to analyze its effectiveness on the improvement of secretion glycoconjugates. BAX was chosensince this protein, as a member of the *bcl*-2 family, it is considered as a stimulating factor for apoptosis [32] a phenomenon strongly affected by oxidative stress.

## 2. Materials and Methods

### 2.1. Animals and Experimental Design

The animal care procedures were in accordance with Legislative Decree No. 146, implementing Directive 98/58/EC of 20 July 1998 concerning the protection of animals kept for farming purposes. The research was approved with Protocol E81AC. 10/A by the Ethics Committee of the University of Camerino.

Thirty-two Duroc x Large White pigs (castrated males; 7 months old), previously randomly divided into two homogenous (as body weight, sex, age, and physiological condition regard) groups of 16 pigs each and housed in a commercial farm in 3 × 3 m pens containing 4 pigs each, were used. The numerousness of animals for each group was calculated and considered optimal for a significance level of 0.05, a test power of 0.8, and an effect size of 1. The animals were fed according to a three-phase feeding program as summarized in Table 1 and Table S1. In particular, as present study concerns, during the finisher stage (3 months; from 120 to 180 kg live weight) the two experimental group were fed: (1) degermed corn-barley-soybean-based diet (CTR group); (2) CTR diet supplemented (2 g/kg) with OAE (EXP group).

The supplementation level of OAE was chosen based on our previous data in swine [1,2] and poultry [8,9,10,11,12,13,14,15,16,17,18,19], to keep the feed cost within acceptable limits and without modifying the meat quality traits and the consumer perceptions of meat quality. Information concerning the OAE extraction and analysis methods adopted were previously described [33] and are summarized in the Supplementary Materials. Water was provided ad libitum and the diets were wet (water to feed ratio of 3:1). The animals were slaughtered (10 months old) in a slaughterhouse in accordance with the European Union regulation on the protection of animals at the time of killing (Council Regulation EC No. 1099/2009). Animal growth performances were analyzed evaluating mean body weight (kg), assessed at the beginning and at ending of finisher stage, average daily gain (g), and feed conversion efficiency.

### 2.2. Morphological and Histochemical Analyses

Samples of the duodenum (4 cm removed from the pyloric sphincter) and ascending colon (in the second outer turn) were collected from thesubjects belonging to the two groups. Duodenum was chosen because shows two different secretory structures (goblet cells and duodenal glands) and it is the intestinal tract receiving the stomach content, while the ascending colon represents the longest tract of large intestine acting as fermentation chamber and hosting the vitamins releasing bacteria. The samples intended for conventional glycohistochemistry were immediately fixed by immersion in Carnoy’s fluid for 24 h followed by post-fixation in 2% calcium acetate and 4% paraformaldehyde solution (1:1) for 3 h at room temperature [27], while the others collected to assess morphological characteristics and to perform immunohistochemistry were promptly immersed in 10% neutral-buffered formalin solution for 24 h and so carefully fixed [34]. All specimens were then dehydrated with a graded ethanol series, cleared in xylene, embedded in paraffin wax, and cut into 5-μm-thick serial sections. The sections were stained with hematoxylin and eosin for the morphological analysis [35]. Carbohydrate characterization was performed by staining with Periodic acid–Schiff (PAS) (evidencing vicinal diols), Alcian blue (AB) pH 2.5 (evidencing acid groups), AB-PAS, AB pH 1 (evidencing sulphated groups), AB pH 0.5 (evidencing highly sulphated groups), low iron diamine (LID) (evidencing acid groups), and high iron diamine (HID) (evidencing sulphated groups) [35]. The specific sugar moieties visualized by the different histochemical treatments are shown in Supplementary Table S2. Adjacent serial sections were pre-treated with Sialidase (Sial, type V from *Clostridium perfringens*) before staining with AB pH 2.5 (Sial-AB). Prior to enzymatic treatment, some sections had been incubated with 1% KOH in 70% ethanol for 20 min at room temperature to remove the acetyl groups (KOH-Sial-AB) [22]; the controls for enzyme effectiveness were exposed to neuraminidase-free buffer under the same experimental conditions.

In order to study oxidative stress, immunohistochemical staining for the BAX protein was performed [36]. Rehydrated sections were treated with 3% H_2_O_2_ in order to quench endogenous peroxidase activity and then irradiated in a microwave oven at 750 Watts in 0.1M citrate buffer, pH 6.0 to retrieve the antigens. The sections were then blocked with normal goat serum (Vector Laboratories, Burlingame, CA, USA) for 30 min and incubated overnight with 1:100 mouse monoclonal anti-BAX antibody (sc-7480; Santa Cruz Biotechnology, Santa Cruz, CA, USA). The following day, the sections were incubated with 1:200 biotin-conjugated goat anti-mouse secondary antibody (BA9200; Vector Laboratories, Burlingame, CA, USA). The immunohistochemical reaction was visualized using an avidin-biotin system (PK6100; Vectastain ABC kit; Vector Laboratories) and diaminobenzidine (DAB) as chromogen (SK4100; Vector Laboratories). Some sections incubated with PBS and/or normal goat IgG instead of the primary antibody were used as controls of unspecific staining. All sections were observed under a photomicroscope (Nikon Eclipse E800, Nikon Corp., Tokyo, Japan). For each animal, three independent observers, unaware of the treatments carried out, evaluated five microscopic fields of each intestinal tract and the intensity of the staining was graded in numerical units as follows: negative (0), weak (0.5), moderate (1) and strong (2).

### 2.3. Statistics

Concerning the histochemical data, numerical units of reactivity, sorted in a scale ranging from 0 (negative) to 3 (strong), were converted into ranks [19,20,21,22,23,24,25,26,27,28,29,30,31,32,33,34,35,36,37]. Wilcoxon Mann–Whitney tests were performed: (1) for each histochemical treatment, under the null hypothesis that the distributions of reactivity ranks of the two dietary groups, differ by a location shift of zero; (2) for each diet group, under the null hypothesis that the distributions of reactivity ranks between the serial treatments AB pH 2.5, Sial-AB, and KOH-Sial-AB differ by a location shift of zero. The statistical elaborations were performed using the R version 3.0.2 (R Core Team, 2013) and the stats R-package (wilcox.test function).

## 3. Results

CTR and EXP groups did not show differences in growth performances, as reported in Supplementary Table S3.

The morphological features of both duodenum and colon were well preserved and no appreciable differences between the two groups were observed at light microscopy. In short, the duodenum tunica mucosae appeared raised in villi covered by a simple columnar epithelium with scattered goblet cells; at the base of the villi the epithelium forms small, deep, glandular crypts; duodenal glands were observed in the tunica submucosa (Supplementary Figure S1). The colon is characterized by the glandular crypts which appear deeper and showing more goblet cells than the duodenum ones (Supplementary Figure S1).

**Figure 1:**
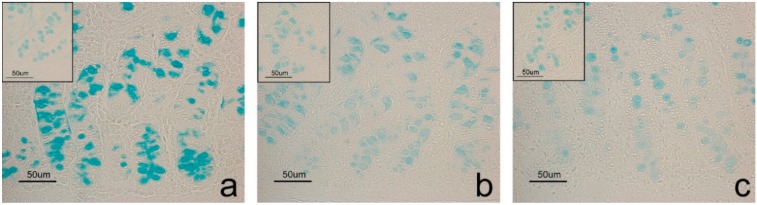
Mucosal duodenum sections derived from EXP samples. Images show differentiated AB reactivity (**a**) of goblet cells induced by Sial-AB (**b**) and KOH-Sial-AB (**c**) histochemical treatments that result in a decreasing reaction intensity. Insets in each image show the reactivity to the same histochemical treatments of CTR samples in which Sial and KOH-Sial sequential treatments do not modify the AB reactivity.

**Figure 2:**
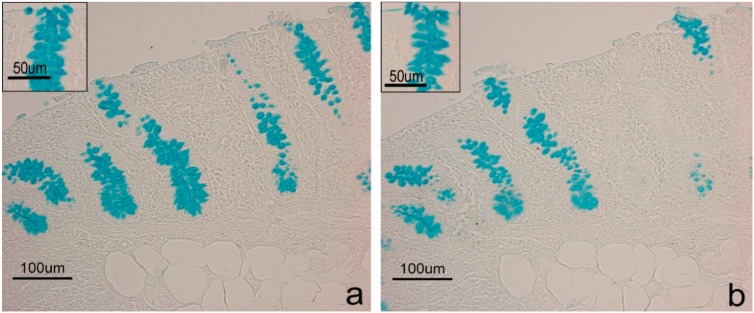
Colon sections derived from EXP samples. Images show differentiated AB reactivity (**a**) of goblet cells induced by Sial-AB; (**b**) histochemical treatment producing the disappearance of AB binding sites. Insets in each image show the reactivity to the same histochemical treatments of CTR samples.

**Figure 3:**
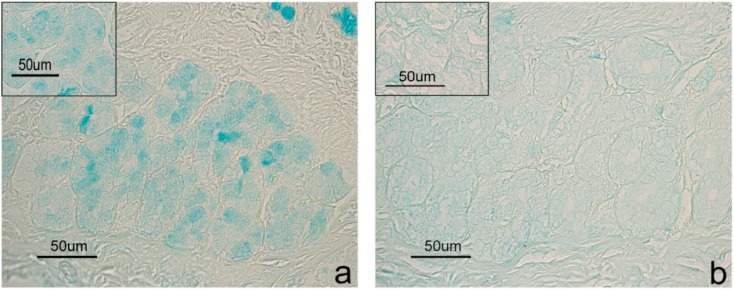
Duodenal glands from CTR samples. The sequential AB (**a**) and Sial-AB (**b**) histochemical treatments produce a decrease of reaction intensity. Insets in each image show the reactivity to the same histochemical treatments of EXP samples.

Positive histochemical results were obtained at the goblet cell level in both duodenum and colon, and at duodenal glands. Differences emerged between CTR and EXP groups concerning histochemical treatment response. The response to the Sial-AB and KOH-Sial-AB histochemical treatments, which induced differentiated reactivity of goblet cells in both duodenum (Figure 1) and colon (Figure 2) of the samples from pigs fed the EXP diets, showed the most evident difference. Contrastingly, the duodenal glands showed the same response to the sequential AB, Sial-AB treatments in both CTR (Figure 3) and EXP groups. As regards the goblet cells, samples from CTR and EXP groups showed significantly different reactivity to AB pH 1, AB pH 0.5, and HID in both duodenum and colon, while LID positivity was significantly different only between duodenal samples. After AB/PAS treatment, duodenal goblet cells were AB reactive at the villus apical portion; while a different picture was observed at the base of villi goblet cells, as they were prevalently AB positive in CTR samples and AB/PAS reactive in the EXP group samples. The incubation of sections in enzyme-free buffer solution resulted in unmodified AB reaction (Supplementary Figure S2).

The responses of the samples, as reactivity intensities, to the various glycohistochemical treatments in duodenum and colon, are shown in Table 2 and Table 3, also indicating the significance of differences between the diet groups for each histochemical treatment. The absolute values of *p* for each histochemical treatment are reported in Supplementary Tables S4 and S5.

The significance of the differences among the serial treatments AB pH 2.5, Sial-AB, and KOH-Sial-AB for CTR and EXP groups are shown in Table 4.

Based on the histochemical evidence, the variety of glycoconjugates produced by duodenal and colon secretory structures are illustrated in Table 5 in descending semi-quantitative order. It should be taken into account that the specific glycoconjugate types are mostly identified based on reactivity difference by comparing sequential treatments (i.e., AB at different pH values, LID vs. HID, AB pH 2.5 vs. Sial-AB vs. KOH-Sial-AB, etc.).

**Figure 4:**
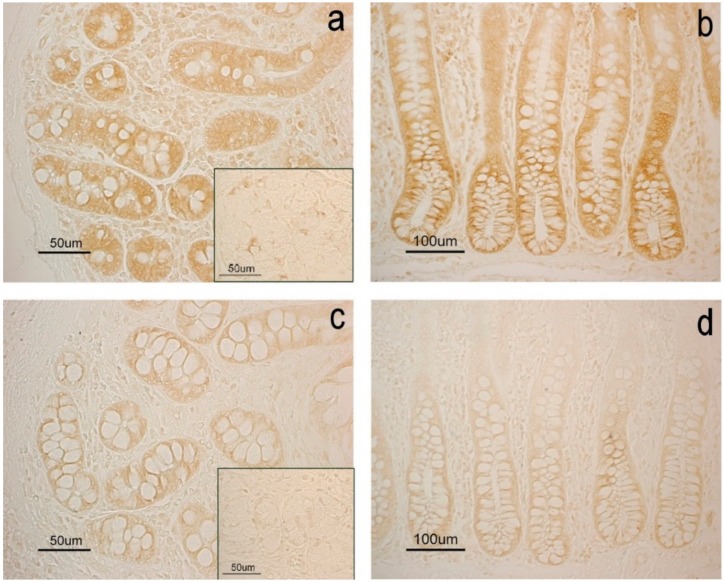
Immunohistochemical reactivity of swine gut. DAB staining is evident in the mucosal intestinal glands of the duodenum (**a**,**c**) and colon (**b**,**d**) and in some duodenal gland cells of the submucosal layer (inserts). Image shows stronger immunohistochemical staining in the CTR group (**a**,**b**) than the EXP group (**c**,**d**).

The immunohistochemistry revealed BAX protein staining in both duodenum and colon. The mucosal epithelium ranged from strong to slightly positive in the colon and the bottom of duodenal villi and it was negative in the apical part of these structures. Immunohistochemical staining was observed in the enterocytes while the goblet cells were always negative. BAX staining was also observed in some scattered cells of duodenal glands. Besides the epithelium, BAX was detected in the vascular endothelium and the neuronal cell bodies of the ganglia localized in submucous and myenteric plexuses. No staining was observed in the negative controls (Supplementary Figure S3). As regards the differences between the two groups, immunohistochemical analysis revealed a lower expression of BAX in the duodenum and colon of the EXP group than in the CTR group (Figure 4); results are summarized in Table 6 also indicating the significance of differences between the diet groups for each histochemical treatment.

## 4. Discussion

Oregano diet supplementation did not produce differences in growth performances respect to that CTR group.

On the basis of reactivity towards the histochemical treatments, it can be affirmed that both the CTR and EXP groups produced large amounts of high sulphated glicoconjugates represented by heparin/heparansulphated-like material and a smaller amount of carboxylated glicosaminoglycans from the goblet cells of both pig duodenum and colon; while duodenal glands produce neutral and sialilated glycoproteins showing sialic acid not acetylated at C_4_ level [22,27,29,35].

OAE diet supplementation promotes the production of sialilated glycoproteins with and without C_4_ acetylated sialic acid in both intestinal tracts under study, especially in the duodenum, where the chondroitinsulphate A/B/C-like glycosaminoglycans production is also induced. No difference in duodenal gland glycoconjugate production was observed between the two groups.

On comparing the glycohistochemical profiles of the EXP and CTR groups, a clear increase in acid glycoconjugate production was observed, which allows for enhancement in water attraction, thus improving tissue hydration and mucosae lubrication. Moreover, the chondroitinsulphate A/B/C-like material increases the negative charge of the viscoelastic barrier produced by the duodenal goblet cells and could act as additional hapten-like sites for pathogen agents; therefore, the duodenal mucous membrane could have indirect defense advantages performed by these additional glycoderivatives, due to both steric hindrance effect and masking action against true pathogen attack sites. A direct defense response is promoted by the production of sialilated glycoproteins by both the duodenal and colon goblet cells in the EXP group; in particular, the C_4_ acetylated sialoderivatives produced by duodenal goblet cells could be able to confer resistance to bacterial sialidase [25,26]. All these defense actions are of paramount importance if we consider that the two examined intestinal tracts perform specific and different functions.

Considering that the goblet cells produce a great variety of glycoconjugates which seemed to be affected by oregano supplementation and that the duodenal glands secrete neutral and sialilated glycoproteins that are not modified by dietary regime, it can be hypothesized that duodenal gland secretion could be involved in buffer and/or enzymatic functions, while secreted products from goblet cells of both duodenum and colon could be mainly involved in lubrication and defense responses.

The above-mentioned considerations suggest that oregano food supplementation enhances both the quality and quantity of the secretion glycoconjugates involved in direct and indirect defense, thus better preserving mucous membranes of pig gut. Finding of the presence of BAX further support this hypothesis; in fact, lower levels of BAX immunostaining intensity were observed in both the studied intestinal tracts in the swine belonging to the EXP group compared with the CTR group.

The BAX protein in the normal human intestine has already been reported [38]; as a member of the Bcl-2 family, it is able to regulate the permeabilization of mitochondrial outer membrane, thus affecting the mitochondrial functionality and, as a consequence, controlling cell apoptosis [39]. Recent studies show that altered functionality of mitochondria can be the cause of intestinal diseases, such as, in humans, colorectal cancer and inflammatory bowel diseases [40]. Therefore, the study of all the factors that can affect the structural and functional integrity of the mitochondria (as well as the diet) can have important implications in the study of intestinal diseases [41,42].

In this study, the reduced expression of BAX protein in EXP group swine supports glycohistochemical results showing an OAE positive action on duodenum and colon mucosae. It can be supposed that the diet supplemented with OAE induced an enhanced anti-oxidative effect on tissues, including ROS generation and cellular constituent damage onset prevention.

## 5. Conclusions

Even if the CTR diet group shows a good situation regarding both the glycoconjugate and BAX expression, the addition of oregano aqueous extract to the diet induces the production of a greater (both quantitatively and qualitatively) variety of glycoderivatives and decreases the BAX presence. Findings allow us to hypothesize that the oregano supplementation strongly enhances the levels of endogenous defense and the antioxidant ability in both duodenum and colon of pig.

## Figures and Tables

**Table 1:** Percentage composition of feed in the three-phase feeding program.

Component	Phase 1 (from 30 to 90 kg)	Phase 2 (from 90 to 120 kg)	Phase 3 (from 120 to 180 kg)
Degermed corn	48	49.92	51
Barley	20.25	21.06	21.53
Wheat	6.75	7.02	7.17
Soybean oil meal	22.15	19.2	16.5
Calcium carbonate	1.25	1.2	1.2
Di-calcium phosphate	0.75	0.75	0.75
Mineral vitamin premix	0.3	0.3	0.3
Sodium chlorine	0.25	0.25	0.25
Lysine	0.25	0.25	0.25
Methionine	0.05	0.05	0.05
Conjugated Linoleic Acid	0	0	1

**Table 2:** Responses of pig duodenum secretory structures.

Pig Duodenum	CTR		EXP		*p*	
Histochemical Treatment	Goblet Cells	Duodenal Glands	Goblet Cells	Duodenal Glands	Goblet Cells	Duodenal Glands
AB pH2.5	1/2	0.5/1	2	0.5/1	*	ns
Sial-AB	1/2	0	1/2	0	ns	ns
KOH-Sial-AB	1/2	0	0.5/1	0	*	ns
AB pH1	1	0	0.5/1	0	*	ns
AB pH0.5	1	0	0.5	0	*	ns
PAS	1/2 ^a^	1	1 ^a^	1	ns	ns
	2 ^b^		2 ^b^		ns	
AB/PAS	B1/R0 ^a^	B0/1/R1	B2/R0 ^a^	B0/1/R1	**	ns
	B2/R1 ^b^		B2/R2 ^b^		*	
LID	1/2	0.5/1	2	0.5/1	*	ns
HID	1	0	0.5/1	0	*	ns

CTR = Degermed corn-barley-soybean-based diet. EXP= CTR diet supplemented with oregano aqueous extract. AB = Alcian blue; Sial = Sialidase; PAS = Periodic acid–Shiff; LID = Low iron diamine; HID = High iron diamine; B = Blue; R = Red; * = *p* < 0.05; ** = *p* < 0.01; ns = not significant. ^a^ = villus apical portion; ^b^ = villus basal portion.

**Table 3:** Responses of pig colon goblet cells.

PIG COLON	CTR	EXP	*p*
Histochemical Treatments			
AB pH2.5	2	2	ns
Sial-AB	2	½	*
KOH-Sial-AB	2	½	*
AB pH1	1	0.5/1	ns
AB pH0.5	1	0.5/1	*
PAS	1	1	ns
AB/PAS	B2/R0	B2/R0/1	*
LID	2	2	ns
HID	1	0.5/1	*

CTR = Degermed corn-barley-soybean-based diet.EXP= CTR diet supplemented with oregano aqueous extract. AB = Alcian blue; Sial = Sialidase; PAS = Periodic acid–Shiff; LID = Low iron diamine; HID = High iron diamine; B = Blue; R = Red; * = *p* < 0.05; ns = not significant.

**Table 4:** Statistical significance values of differences in location shift of reactivity ranks, obtained using Wilcoxon Mann–Whitney tests, between the sequential treatments AB pH 2.5 vs. Sial-AB, and Sial-AB vs. KOH-Sial-AB, inside each diet group (CTR and EXP) in duodenum and colon of pig. The significance threshold was set at *p* = 0.05.

Intestinal Tract	Diet	Secretory Structure	AB pH 2.5 vs. Sial-AB	Sial-AB vs. KOH-Sial-AB
Duodenum	CTR	Goblet cells	0.821	0.821
	EXP	Goblet cells	0.011	0.011
	CTR	Duodenal glands	0.007	1.000
	EXP	Duodenal glands	0.008	1.000
Colon	CTR	Goblet cells	1.000	1.000
	EXP	Goblet cells	0.015	1.000

CTR = Degermed corn-barley-soybean-based diet. EXP= CTR diet supplemented with oregano aqueous extract. AB = Alcian blue; Sial = Sialidase.

**Table 5:** Complex carbohydrates produced by analyzed secretory structures under different dietary treatments.

Diet	Complex Carbohydrates Produced by Duodenal Goblet Cells	Complex Carbohydrates Produced by Duodenal Glands	Complex Carbohydrates Produced by Colon Goblet Cells
CTR	Heparin/heparansulphate-like GAGs Hyaluronic acid/chondroitin-like GAGs	Neutral and sialilated not C_4_ acetylated glycoproteins	Heparin/heparansulphate-like GAGs Hyaluronic acid/chondroitin-like GAGs
EXP	Sialilated glycoproteins with and without C_4_ acetylated SA Heparin/heparansulphate-like GAGs Chondroitinsulphate A/B/C-like GAGs	Neutral and sialilated not C_4_ acetylated glycoproteins	Heparin/heparansulphate-like GAGs Hyaluronic acid/chondroitin-like GAGs Sialilated glycoproteins not C_4_ acetylated

CTR = Degermed corn-barley-soybean-based diet supplemented. EXP= CTR diet supplemented with oregano aqueous extract. GAGs = Glycosaminoglycans.

**Table 6:** Intensity of BAX staining in tissue sections.

IntestinalTract	CTR	EXP	*p*
Duodenum	2	0.5	*
Colon	2	0.5	*

CTR = Degermed corn-barley-soybean-based diet. EXP = CTR diet supplemented with oregano aqueous extract. * = *p* < 0.05.

## Supplementary Materials

The following are available online at https://www.mdpi.com/2076-2615/10/1/149/s1. Table S1: Feed chemical analysis; Table S2: Sugar moieties visualized by performed histochemical treatments; Table S3: Animal performances; Table S4: Absolute *p*-values of histochemical response difference in pig duodenum secretory structures; Table S5: Absolute *p*-values of histochemical response difference in pig colon goblet cells; Figure S1: Light micrograph of duodenum and colon; Figure S2: Controls for enzyme effectiveness; Figure S3: Negative controls for the BAX immunohistochemistry in swine duodenum and colon.
