# Supplementary material for: Oregano Feed Supplementation Affects Glycoconjugates Production in Swine Gut
Source: Animals (Basel). 2020 Jan 16;10(1):149. doi: 10.3390/ani10010149 (PMC7023309; doi:10.3390/ani10010149)
Supplement: Supplementary file 1 [file animals-10-00149-s001.pdf]

Oregano feed supplementation affects glycoconjugates production in swine gut

Francesca Mercati, Cecilia Dall'Aglio, Gabriele Acuti, Valerio Faeti, Federico Maria Tardella, Carolina Pirino, Elena De Felice, Paola Scocco

Material and methods

Extraction and analysis of oregano aqueous extract (OAE)

The OAE was obtained by a process of bio-liquefaction based on enzyme bio-catalysis [1] as previously described by Franciosini et al. [2]. Briefly, the plant material was boiled in water, and treated with a specific enzymatic preparation after cooling for four hours, and finally filtered. The OAE obtained was analyzed to quantify

- antioxidant capacity, measured in terms of radical scavenging ability using the stable radical DPPH [3],
- total polyphenols, evaluated using the Folin-Ciocalteu reagent [4],
- reducing sugars was evaluated using the ADNS method [5].

Table S1. Feed chemical analysis in the three-phase feeding program

Component (%)	Phase 1 (from 30 to 90 Kg)	Phase 2 (from 90 to 120 Kg)	Phase 3 (from 120 to 180 Kg)
Dry matter	86.816	86.518	86.856
Crude Protein	15.537	14.366	14.081
Crude Fat	1.634	1.618	2.703
Fiber	3.366	3.252	3.112
Ash	4.633	4.422	4.891
Starch	49.435	50.547	52.067

Table S2. Sugar moieties visualized by performed histochemical treatments

HISTOCHEMICAL TREATMENTS	SUGAR MOIETIES VISUALISED
AB pH2.5	Acidic groups: Sialic acid (SA), carboxylated (Hyaluronic acid, Chondroitin) and sulphated (Chondroitin-sulphates A/B/C, Heparin, Heparansulphate) Glycosaminoglycan (GAG)-like material
Sial-AB	Acidic groups without C_4 not acetilated SA
KOH-Sial-AB	Asialilated acidic groups (GAG-like material)
AB pH1	Sulphated GAG-like material (Chondroitin-sulphates A/B/C, Heparin, Heparansulphate)
AB pH0.5	Highly sulphated GAG-like material (Heparin, Heparansulphate)
PAS	Vicinal hydroxyls (neutral and sialilated glycoproteins, GAG-like material)
AB/PAS	Acidic groups and vicinal hydroxyls
LID	Acidic groups
HID	Sulphated GAG-like material

AB = Alcian blue; Sial = sialidase; PAS = periodic acid Schiff; LID = low iron diamine; HID = high iron diamine

Table S3. Animal performances

GROWTH PERFORMANCE	CTR DIET	EXP DIET	*p*
Mean Body weight at beginning of Finisher stage (Kg)	120.2	119.8	n.s.
Mean Body weight at ending of Finisher stage (Kg)	183.9	184.2	n.s.
Average Daily Gain (g)	729	748	n.s.
Feed Conversion Efficiency	4.05	3.85	n.s.

CTR: degermed corn-barley-soybean-based. EXP: CTR diet supplemented (2 g/kg) with an oregano aqueous extract. $P < 0.05$; n.s.: not significant

Table S4. Absolute P values of histochemical response difference in pig duodenum secretory structures

PIG DUODENUM		*p*	
Histochemical treatment		Goblet cells	Duodenal glands
AB pH2.5		0.01116	1
Sial-AB		0.9336	-
KOH-Sial-AB		0.01066	-
AB pH1		0.01471	-
AB pH0.5		0.01066	-
PAS	a	0.8294	-
	b	0.1425	
AB/PAS	a	0.007937	1
	b	0.01167	
LID		0.01066	1
HID		0.01018	-

AB = Alcian Blue;Sial = Sialidase; PAS = Periodic Acid Shiff; LID = Low Iron Diamine; HID = High Iron Diamine. [a] = villus apical portion; [b] = villus basal portion

Table S5. Absolute *P* values of histochemical response difference in pig colon goblet cells

PIG COLON Histochemical treatments	*p*
AB pH2.5	1
Sial-AB	0.01193
KOH-Sial-AB	0.01193
AB pH1	0.05701
AB pH0.5	0.0144
PAS	1
AB/PAS	0.01167
LID	1
HID	0.01471

AB = Alcian Blue; Sial = Sialidase; PAS = Periodic Acid Shiff; LID = Low Iron Diamine; HID = High Iron Diamine.

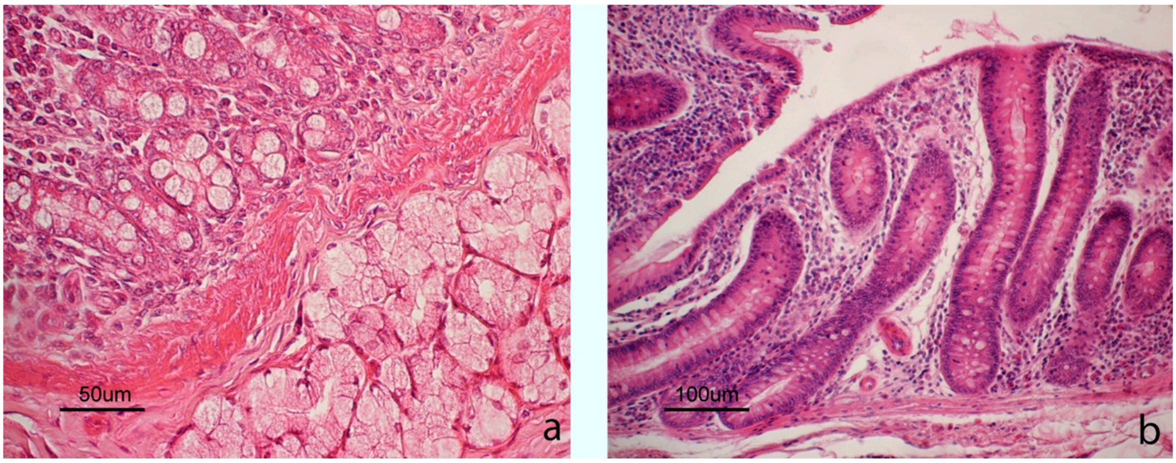

Figure S1 Light micrograph of (**a**) duodenum and (**b**) colon. In the duodenum, glandular crypts and serous duodenal glands in the tunica submucosa are showed. In the colon, deep glandular crypts are evident. Hematoxylin-eosin staining.

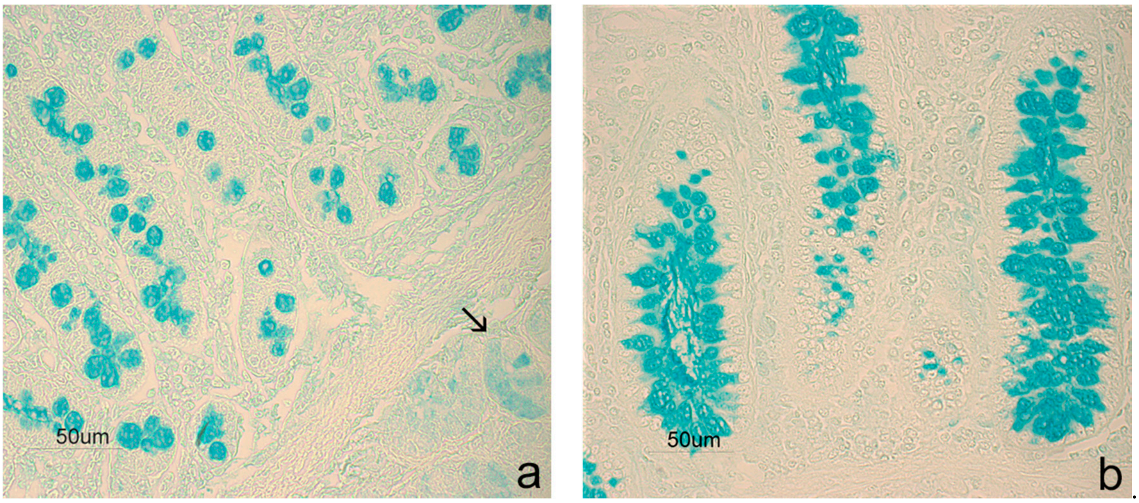

Figure S2. Controls for enzyme effectiveness. Sections incubated with enzyme-free buffer showed AB strong reactivity in swine duodenal goblet cells (**a**) and submucosal glands (↑), as well in colon goblet cells (**b**).

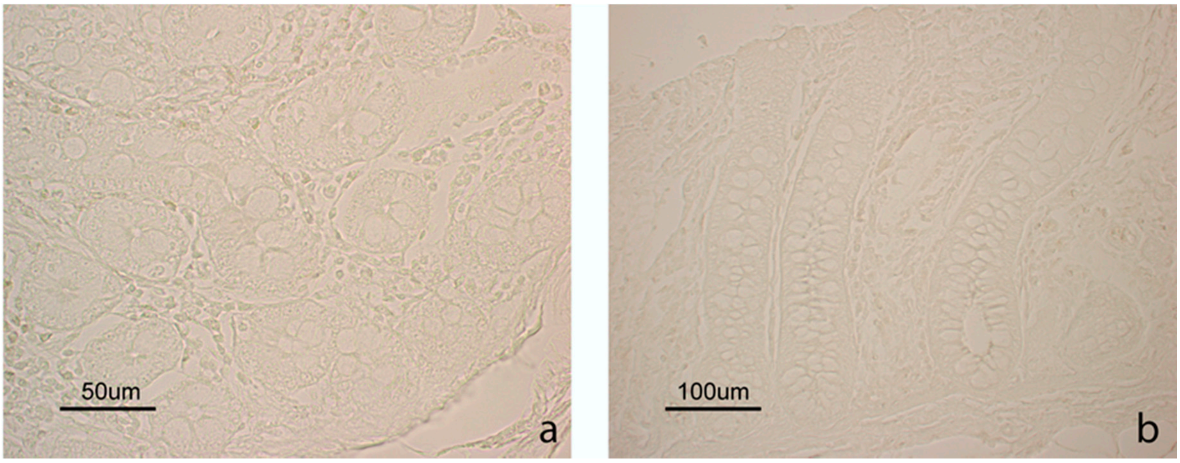

Figure S3. Negative controls for the BAX immunohistochemistry in swine (**a**) duodenum and (**b**) colon.

References

1. Setti, L.; Zanichelli, D. Bioliquefaction as a bio-refinery's approach for the production of natural bioactive compounds for functional cosmetics. In: Morselli L, Passarini F, Vassura I, editors. Waste recovery: strategies, techniques and applications in Europe. Italy, Milano: Franco Angeli; **2009**, 122–128.
2. Franciosini, M.P.; Casagrande-Proietti, P.; Forte, C.; Beghelli, D.; Acuti, G.; Zanichelli, D.; Dal Bosco, A.; Castellini, C.; Trabalza-Marinucci, M. Effects of oregano (*Origanum vulgare* L.) and rosemary (*Rosmarinus officinalis* L.) aqueous extracts on broiler performance, immune function and intestinal microbial population. *J. Appl. Anim. Res.* **2016**, *44(1)*, 474–479.
3. Donglin, Z.; Yasunori, H. Phenolics, ascorbic acid, carotenoids and its antioxidant activity of broccoli and their changes during conventional and microwave cooking. *Food Chem*. **2004**, *88*, 503–509.
4. Ainsworth, E.A.; Gillespie, K.M. Estimation of total phenolics content and other oxidation substrates in plant tissues using Folin-Ciocalteu reagent. *Nat Protoc*. **2007**, *2*, 875–877.
5. Bailey, M.J.; Biely, P.; Poutanen, K. Interlaboratory testing of methods for assay of xilanase activity. *J. Biotechnol.* **1992**, *23*, 257–270.
